# The Impact of the COVID-19 Pandemic on China's Manufacturing Sector: A Global Value Chain Perspective

**DOI:** 10.3389/fpubh.2021.683821

**Published:** 2021-05-14

**Authors:** Yuegang Song, Xiazhen Hao, Yilin Hu, Zhou Lu

**Affiliations:** ^1^School of Business, Henan Normal University, Xinxiang, China; ^2^NPD Information Consulting (Shanghai) Co., Ltd., Shanghai, China; ^3^School of Economics, Tianjin University of Commerce, Tianjin, China

**Keywords:** COVID-19 pandemic, GVC reconfiguraiton, GTAP model, manufacturing sector, China

## Abstract

This paper, based on the notion of Trade in Value Added (TiVA), combines the global trade analysis project (GTAP) model with the value-added model in seeking to simulate and assess the impact of the COVID-19 pandemic on China's manufacturing sector in global value chain (GVC) reconfiguration. The empirical study provides three major results. First, at the macroeconomic level, the pandemic wreaks a negative impact on all the economies, including the U.S., in regard to import & export trade, GDP and social welfare policy. Second, nation-level simulation shows that there's a remarkable disparity across different pandemic scenarios in the level of division of labor and of GVC participation for China and its trade partners. Third, sector-level analysis shows that the impacts of the pandemic include promoting the level of GVC participation and of labor division in China's manufacturing sector (electromechanical equipment and computer goods). This paper also provides policy advice for Chinese government: participation in higher-end GVCs, introduction of further structural reforms and retention of foreign investors, and active responses to GVC reconfiguration and cross-border capital flow.

## Introduction

Economic globalization has made the world smaller. With the increasing flow of trade, capital and labor, the COVID-19 pandemic has quickly proliferated across the world, the most remarkable difference when compared with any previous public health crisis at the world level. As the pandemic spreads, more and more countries have implemented border closures in order to effectively contain virus transmission. However, the pandemic hit a vast number of economies hard with significantly delayed business recovery and damaged production network. A multitude of sectors are facing a shortage of supply which dislocates the upstream and downstream supply chains, thereby bringing an impact on the global value chains (GVC), supply chains, trade & investment which accelerates the trend of anti-globalization (1). In consideration of medical supply security and less reliance for foreign assistance, many countries have implemented manufacturing revitalization strategy while retracting overseas investment to the domestic market. These measures might bring significant change to the existing system of global economy and further dim the prospects of globalization (2). Using real trade openness instead of nominal trade openness Gozgor (3) recalculated the KOF economic globalization index from 1970 to 2013, and concluded that economic globalization has a positive effect on economic growth. GVC reconfiguration in the context of anti-globalization will certainly exerts heavy impact on the Chinese economy.

The COVID-19 crisis makes countries to rethink their role in the GVCs and the associated risks. Furthermore, it is expected to accelerate GVC reconfiguration because post-pandemic GVC configuration takes into account not only cost factors; in addition, some countries and multinational corporations are considering a transition to economic detachment and the self-developed value chains, leading to GVC instability and the resulting risk of GVC relocation. GVC localization and regionalization are looming as two prominent factors. As the largest manufacturing economy in the world, the value added of China's manufacturing sector reached 26.9 trillion in 2019, accounting for 28.1% of the global total. As China moves steadily to the upper end of the GVCs, the proliferating pandemic will unavoidably exerts a serious impact on the intra-industry GVC division of labor. Then will the pandemic exert some impact on GVC reconfiguration? If the answer is in the affirmative, how the impact on China's value chain reconfiguration can be measured? How the impacts on China's level of GVC participation and on the country's role in labor of division can be measured across different industries? What policy responses should China take to these impacts? The answers to the questions help China play its due role in GVC reconfiguration. Therefore, it's of great significance to precisely define the pandemic's impact on China's role in GVCs.

The existing investigations deal primarily with the impact of the severe public health crises on regional economic growth and the impact of the COVID-19 pandemic on the global and Chinese economies.

Most of recent studies believe that the impact of the outbreak of major public health emergencies on the global economy or regional economic growth tends to be temporary, although negative. Based on the historical epidemiological economic data of the British flu, Keogh-Brown and Smith (4) built a compact model, finding that if the 1957 or 1968 flu recurred, they would have only a temporary economic impact, causing the British GDP to suffer 3.35% loss in the quarter from the outbreak and a 0.58% loss for the whole year. Verikiosa et al. (5) built a Modified Monash Model (MMM) to assess the impact of swine flu on the Australian economy and found that in spite of the massive investment intention curtailments and the consumer market slumps, the long-term effect on the regional economy remains yet to be established. Bloom and Mahal (6) collected the data about 51 developing economies and industrialized economies for a study of the correlation between AIDS prevalence and per capita GDP growth; the empirical study affirms that an AIDS epidemic will retard economic growth. In contrary, Brainerd and Siegler (7) conducted empirical research on the impact of the Spanish flu on the U.S. economy and, based on the empirical data, established that the disease contributed positively to the economy of the states. Prager et al. (8) studied the impact of a potential flu pandemic on the overall U.S. economy, finding that GDP loss can be effectively lessened by virtue of the government's preventative control measures, e.g., vaccination.

The global COVID-19 pandemic has attracted broad attention from researchers in regard to its impact on the world economy, the preventative control policies made by different countries and the differential effects. McKibbin and Fernando (9), Alvarez et al. (10), Jones et al. (11), and Baker et al. (12) studied the pandemic's impact on the global economy, the balance between pandemic containment and economic performance, and the serious impact of transmission uncertainties on the economic activity. Fornaro and Wolf (13) and Shahbaz et al. (14) proposed the economic depression as a result of the pandemic which will not only lead to a global supply & demand crisis, but also impact heavily on regional employment, productivity growth and foreign direct investment. As states and GVC restrict each other during the COVID-19 pandemic, attention should be paid to GVC structure, states and their interactions (15). From the perspective of a sharp shortage of medical supplies, Gereffi (16) pointed out that during the COVID-19 pandemic the U.S. shortage of N95 respirators is a policy failure more than a market failure. From the perspective of Gourinchas (17), the pandemic's negative impact will loom large in many ways, including corporate supply chain disruption, labor shortage, shutdowns, close-downs, intensely shrinking consumer demand, and credit crunches. Baldwin and Mauro (18), Brightman and Treussare (19), and Ayittey et al. (20) argue that the pandemic's negative impact on the global economy is looming increasingly in the form of global supply chain disruption and trade restriction and the transmission rate exerts a tremendous impact on GVC stability.

Some scholars conducted research on the pandemic's impact on the Chinese economy. Liu (21) carried out a profound analysis of the dynamics of economic globalization in the wake of the pandemic from the perspective of GVC reconfiguration. Zhi and Luo (22) investigated in detail the pandemic's impact on the Chinese economy in both the long term and the short term. Liu (23) sorted out and dissected the characteristics of the pandemic's impact on the Chinese economy and the associated risks while advising on policy-making by pointing out the pandemic does more harm to the producer services sector than to the consumer services sector. Tong et al. (24) analyzed the impact of the proliferating global plague on the Chinese economy and the global economy as well as the countermeasures in response. Wen et al. (25) ascertained that the strict city closures implemented in China after the outbreak wreaks a direct impact on business and extensive close-downs while driving down capacity utilization, level of investment and consumer demand. The strict city closures have worsened China's trade environment and are not conducive to foreign direct investment (26, 27). Some scholars pointed out that in the post-epidemic era, to improve China's position in the global value chain, we must attach importance to technological innovation and improve the quality of export products (28, 29). Zhou et al. (30) employed the econometric ridge regression model to conduct a predictive analysis of the impact sustained by the 2020 growth rate of the Chinese economy. The findings show the vast part of the impact mostly occurred in the first and second quarters before diminishing.

To sum up, literature relevant to the pandemic's impact on the economy agrees that preventative control measures intended to effectively control the extent of the pandemic and lessen its negative impact may pose tremendous potential challenges to a multitude of sectors, e.g., supply & demand, production and financing. Compared with the existing studies, this paper conducts a quantitative analysis based on the GTAP 10 database which is extended to 2020 using Walmsley's dynamic recurrence method. In addition, this paper combines the global trade analysis project (GTAP) model with the value-added model in seeking to simulate and assess the impact of the COVID-19 pandemic on China's manufacturing sector in global value chain (GVC) reconfiguration. The contribution of this paper mainly includes the following three components. First, the author conducts an in-depth, systematic study of the economic impact of the outbreak of public health emergencies, especially epidemics. Particularly, the author builds an accurate computable general equilibrium (CGE) model to assess to what extent the pandemic affects China's GVC participation. Second, based on the aforesaid theoretical findings the author introduces model calibration and model linking to the pandemic to complete data processing for China's GVC participation. Third, the aforesaid linkage model is used for policy simulation and simulation results presentation to interpret the pandemic's effects on China's GVC participation, on both the state level and the industry level.

As for the outline of the subsequent content of the paper, Part 2 analyzes primarily the pandemic's impact on Chinese manufacturing in regard to GVC reconfiguration. Part 3 introduces the linkage model and constructs indices by elaborating on how to link the global trade analysis project (GTAP) model with the decomposition of trade in value added (31) and construct such core indices as forward GVC participation, backward GVC participation, GVC participation and division of labor. Part 4 presents the database and scenarios. Part 5 includes an in-depth simulation-based interpretation of the pandemic's effects on Chinese manufacturing in regard to GVC reconfiguration in such dimensions as the Chinese economy and China's location in division of labor in GVC participation. Part 6 deals with the research and policies.

## COVID-19 Pandemic and GVC Reconfiguration

Currently, GVCs are faced with two major challenges, the pandemic and global trade dispute. Particularly, the U.S.–China trade war in combination with the pandemic has contributed to the world's major economic uncertainty, which might threaten the GVCs. What causes China's role in GVC participation so vulnerable to these challenges?

**Figure 1:**
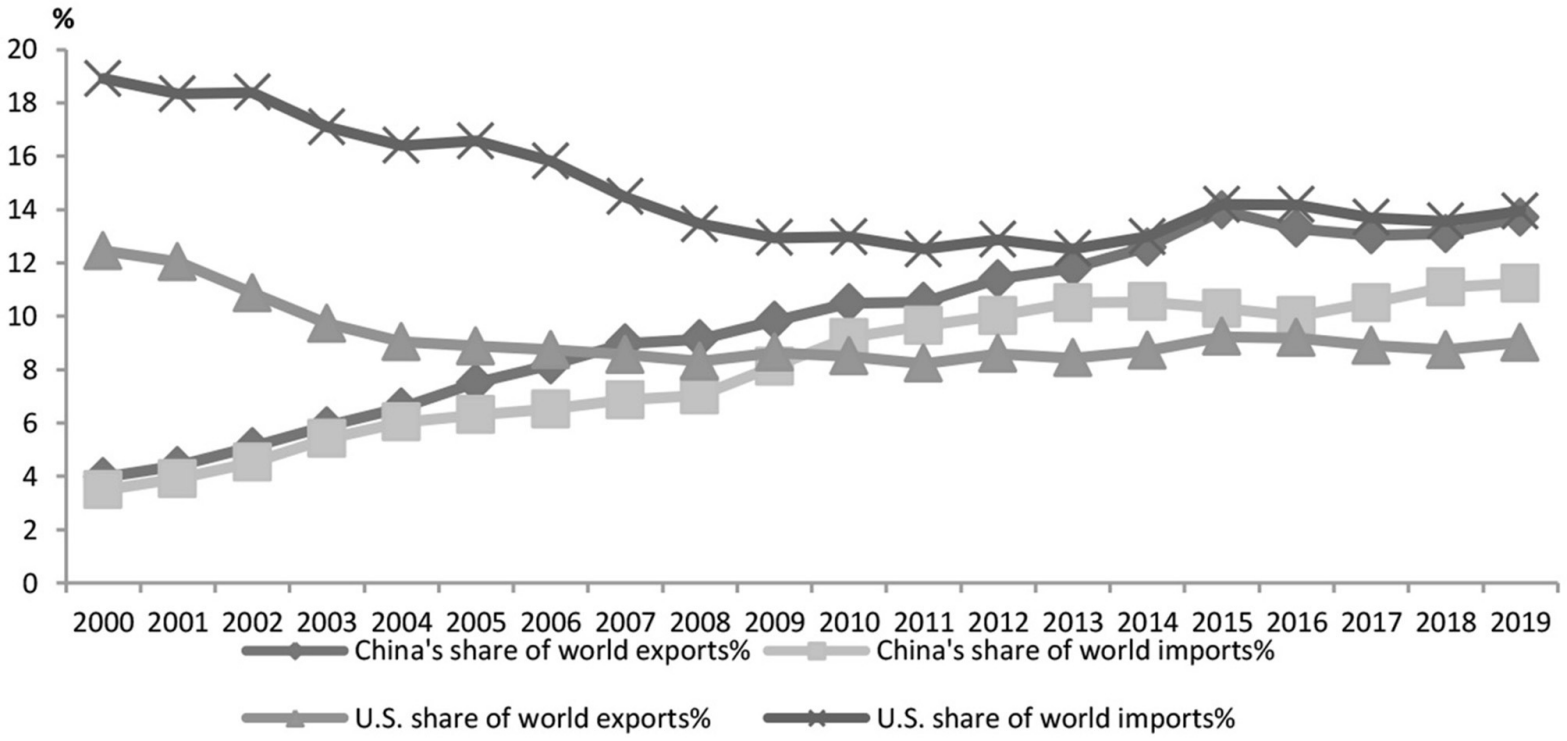
Changes in China and the United States' share of global imports and exports. Data source: UN Comtrade Database.

**Figure 2:**
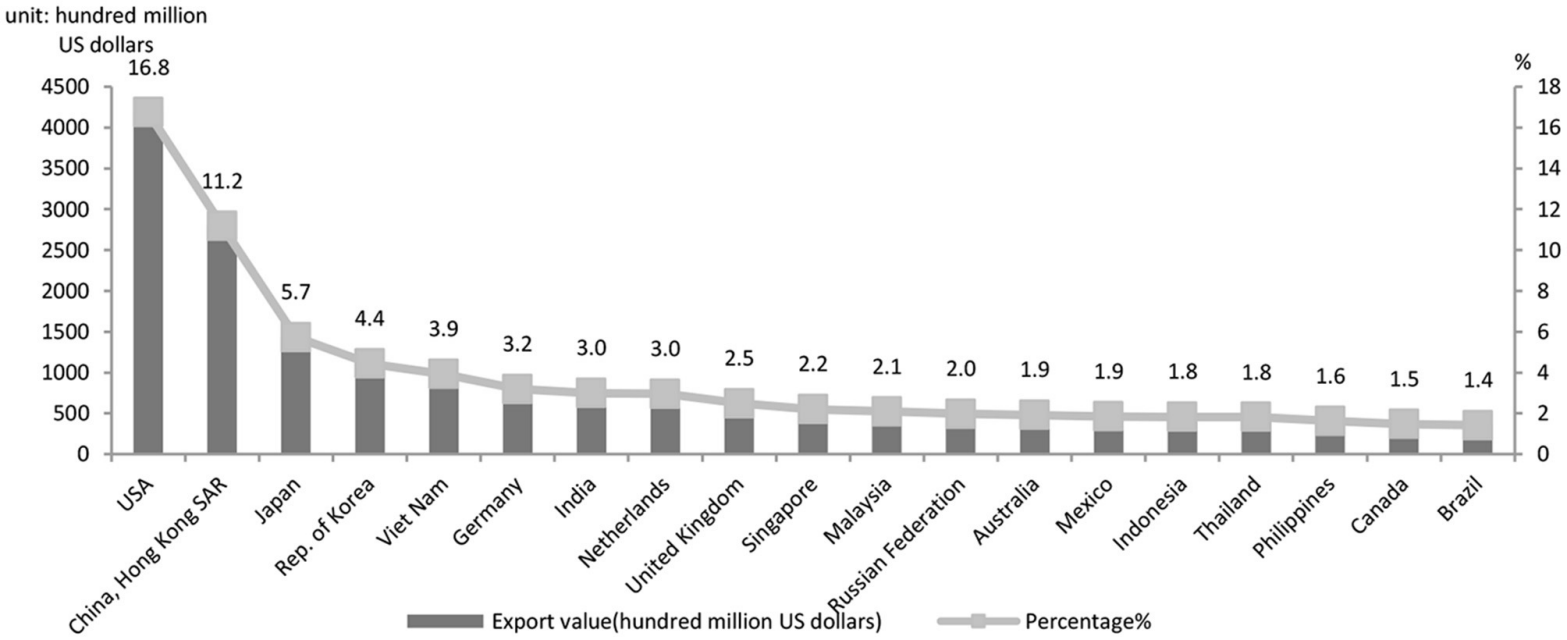
China's export partners in 2019. Data source: WITS.

**Figure 3:**
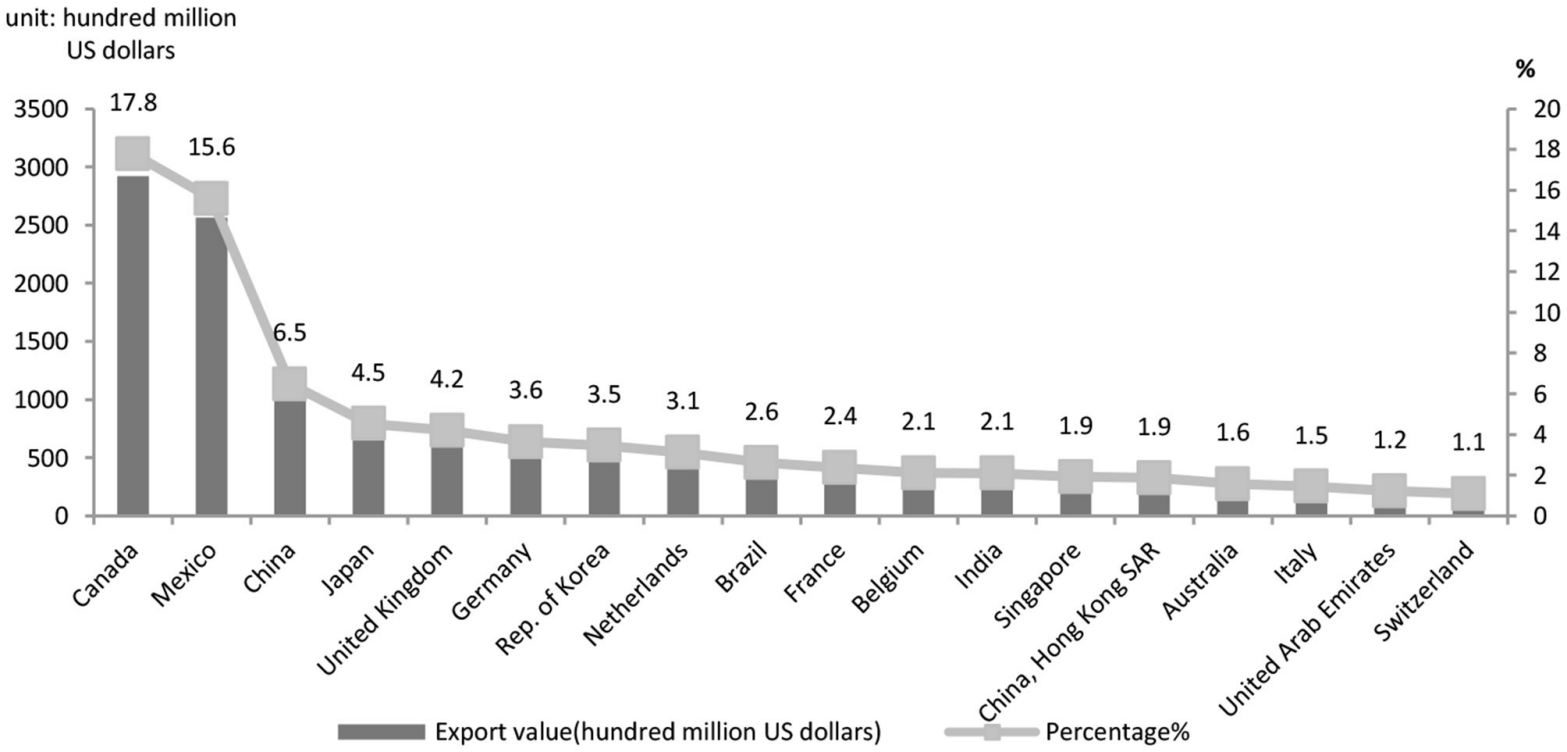
U.S. export partner countries in 2019. Data source: WITS.

First, integration into the GVCs makes China more vulnerable to external impacts. The country's exports account for about 20% of its GDP, indicating its deep integration into the GVCs (UN Comtrade Database, 2020). The goods exported from the country reached the value of $4.576126 trillion, accounting for 13.2% of the world's total, an increase of 0.4% over 2018. There was a steadily-growing share in export on the international market (Figure 1); goods import accounted for 10.8% of the world's total, soaring to a historic high (UN Comtrade Database, 2020). Besides, there was a rise in China's share of the international market (Ministry of Commerce, 2020). In 2019, China's trade with the U.S. totaled $4.1435.8 trillion in value, a 1.5% annual drop as the following statistics show. As shown in Figure 2, the U.S., EU and Japan, major trade partners of China, suffer heavily from the pandemic. Hence the global demand for Chinese capital goods and intermediate goods suffers a significant negative shock. The initial outbreak of the pandemic has resulted in a sharp and intense fall of export of Chinese goods as well as a break of the value chains. The paralysis of the global production network is followed by GVCs and supply chain reconfiguration that deliver a negative impact on international trade (32). Conversely, the data on the U.S. export quota (Figure 3) indicate that in 2019 the U.S. exported 6.5% of its goods to China, its third largest trade partner, larger than the trade partners including the UK, Germany and Brazil which are also hit hard by the pandemic. It is shown in Figures 2, 3 that due to the high interdependence between the Chinese economy and the U.S. economy, the worsening of the stagnant U.S. economy and the U.S.–China trade friction wreaks a tremendous impact on the Chinese economy, now deeply integrated in the GVCs.

Second, China remains mired in the mid-and low-end of the GVCs. Hong and Zhong (33) studied participation of multiple countries in the GVCs using the UNCTAD-Eora GVC Database, suggesting that China remains at the lower end (downstream) of GVC participation in regard to division of labor. At present, China ranks on the third of four levels of global manufacturing, a situation supposedly impossible to be fundamentally improved in the short term (Table 1).

**Table 1:** The four levels of global manufacturing.

**Level**	**Type of manufacturing**	**Country**
1st level	Global technological innovation centers (e.g., the U.S.)	The U.S.
2nd level	High-end manufacturing	EU, Japan, the UK, etc.
3rd level	Mid-and low-end manufacturing	China, Southeast Asia, Brazil, India, etc.
4th level	Natural resource export	OPEC (Organization of the Petroleum Exporting Countries), Africa, Latin America, etc.

*Data Source: Minister of IIT Miao Wei's interpretation of Chinese Manufacturing 2025 at the 13th session of the Permanent Committee of the 12th CPPCC (Dec. 9, 2020; https://dy.163.com/article/FDL3M3ST0539AGEJ.html)*.

**Figure 4:**
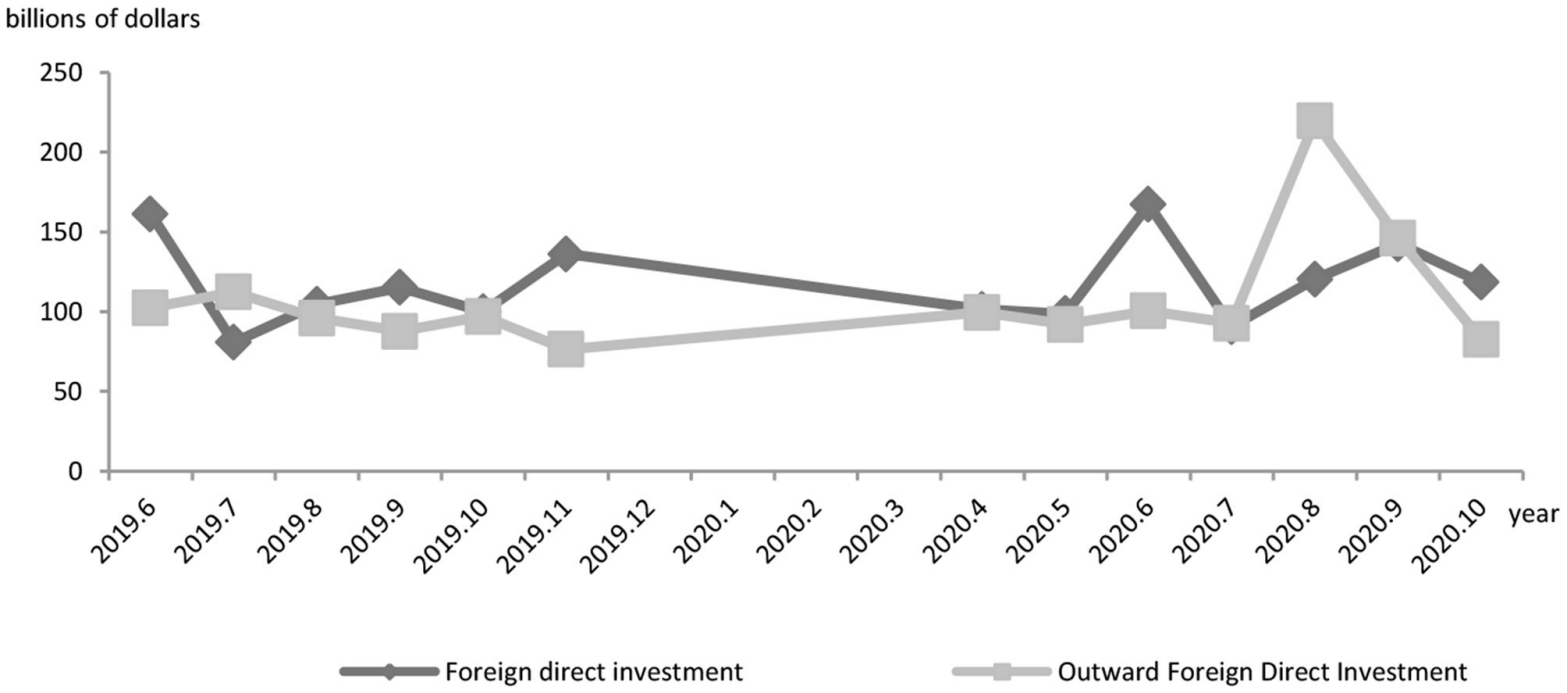
China's foreign direct investment and outward foreign direct investment. Data source: Ministry of Commerce of the people's Republic of China.

Third, the pandemic in combination with the U.S.-China trade friction exposes China to the risk of detachment from the GVCs. The outbreak of the pandemic, combined with the U.S.-China trade friction, wreaks a tremendously negative impact on China's value chains and inward/outward FDI. First, as shown in Figure 4, the pandemic has brought a negative impact on China in regard to FDI-based GVC participation. Notably, the 1-month-long economic stall in favor of disease control affected the China-based foreign-owned companies to rather great a degree. From January to April, 2020, only a $41.34 billion share of the FDI materialized, dropping by 8.4% year on year. What's more, the OFDI (Outward Foreign Direct Investment) activities of Chinese multinationals are affected by the proliferation and transmission of the pandemic at more than one location across the globe. Investment climate change, factor mobility stagnation, market expectations change, etc. have impeded China's FDI activities. The statistics released by the Ministry of Commerce and the State Administration of Foreign Exchange show that China aggregated $64.17 billion in OFDI from January to July, 2020, a 6.5% year-on-year fall. The amount include a $60.28 non-finance OFDI in 4,625 foreign enterprises in 161 economies around the globe, reflecting a 5.4% fall from the preceding year as well as a hindrance to the Go Global initiative which targets participation in GVC reconfiguration.

**Figure 5:**
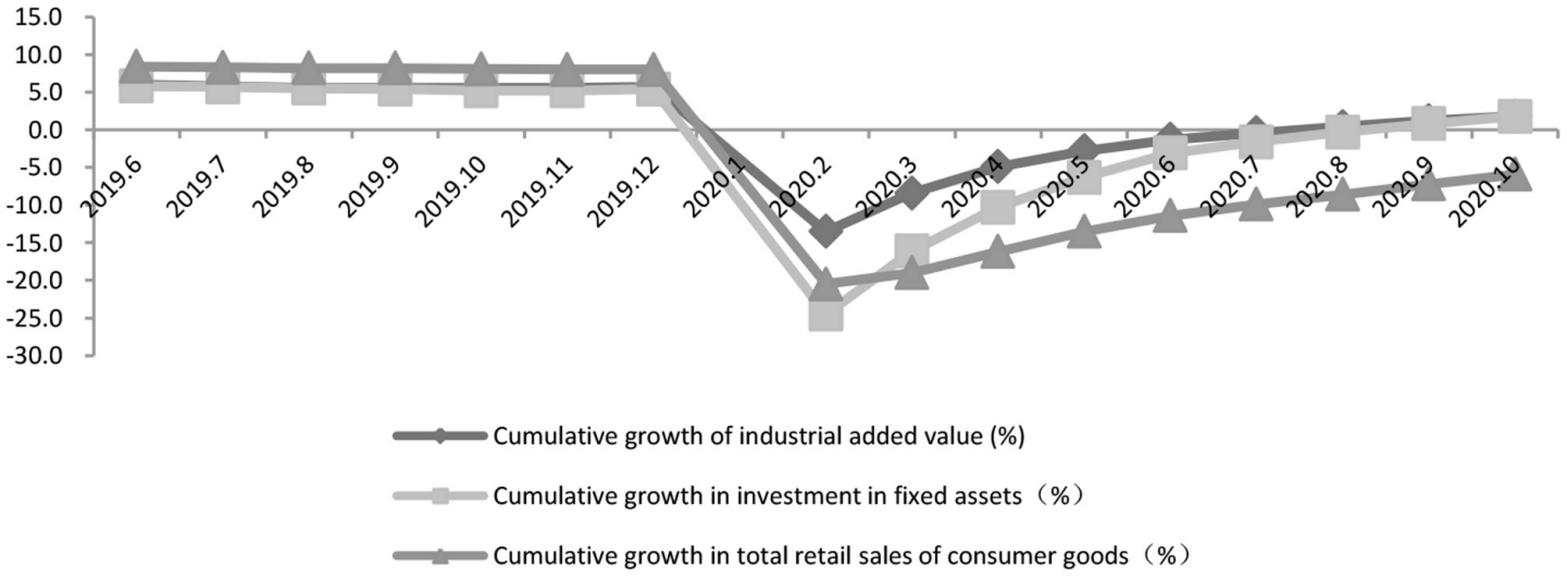
China's industrial value added, investment and social marketing since the outbreak of the COVID-19. Data source: National Bureau of statistics of the People's Republic of China.

**Figure 6:**
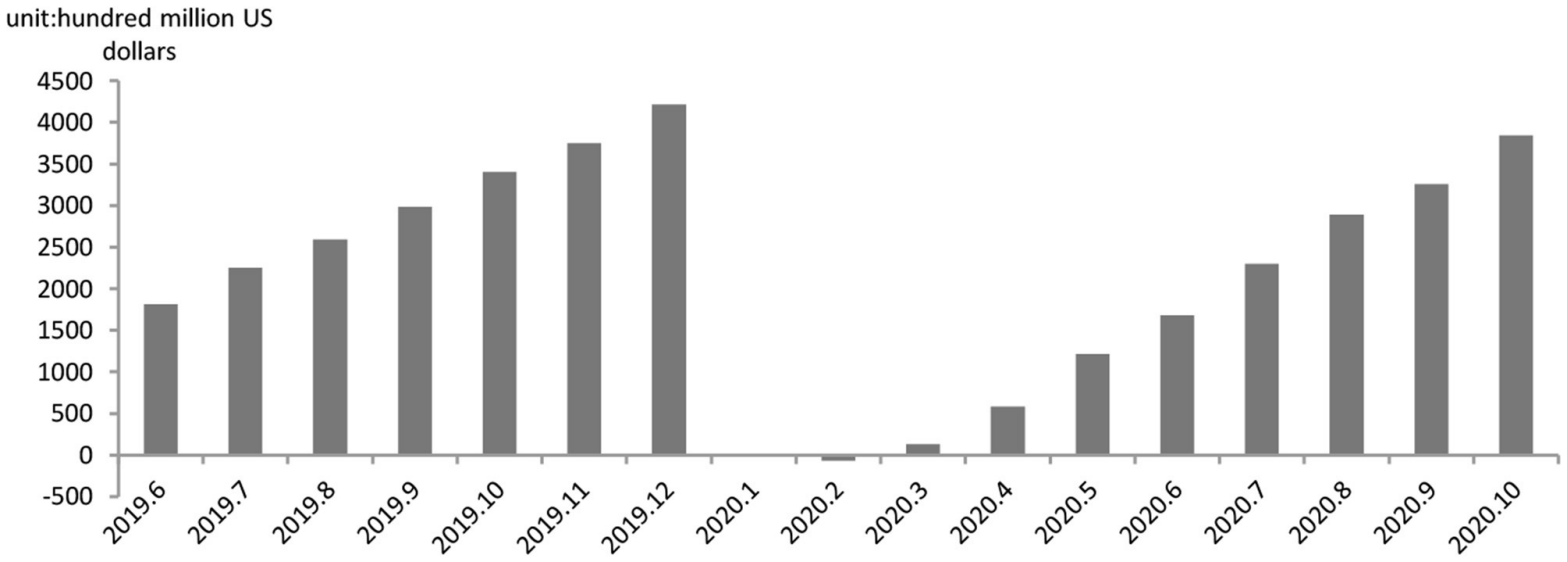
Cumulative value of China's import and export balance. Data source: National Bureau of statistics of the People's Republic of China.

We consider the impacts of the Covid-19 pandemic on China's manufacturing sector in GVCs from two perspectives, supply and demand. The first wave of the pandemic exerted remarkable negative impact on China's production network and brought in export slumps. The outbreak of the pandemic in China has caused the postponement of many orders due to closures of logistics services and shutdowns, therefore reducing demand for intermediate goods as well as export to the U.S. and Europe. Manufacturing's GVCs, therefore, have sustained dislocation. The impact of the pandemic on China's industry value added (IVA), fixed investment (FI) and consumer goods retail sales assumes a V-like trend, industry value added slumping by 13.5%, the FI slumping by 24.5% and consumer goods sales slumping by 20.5% (Figure 5). The slumps mark the first $6.876 billion trade deficit in history (Figure 6). However, the prompt and effective steps taken to contain the virus have resulted in the revival of the production network.

By contrast, the second wave has affected China's foreign trade and its role in GVCs on the demand side. Unlike the 2008 financial tsunami, the COVID-19 pandemic hit China's foreign trade, resulting in not only foreign demand slumps, but also risks of supply chain disruption on the supply side. Besides, the Chinese trade sector is something like post-trade processing which characterizes the commodity structure of both import trade and export trade. In the short run, the impact sustained by China on export seems to be greater than that on import due to the severity of the pandemic in other countries; in the long run, however, the exposure of the world economy to a long downturn will come with a sharp landslide of foreign demand, impeding intermediate goods import and adding uncertainties to the future trade balance.

**Figure 7:**
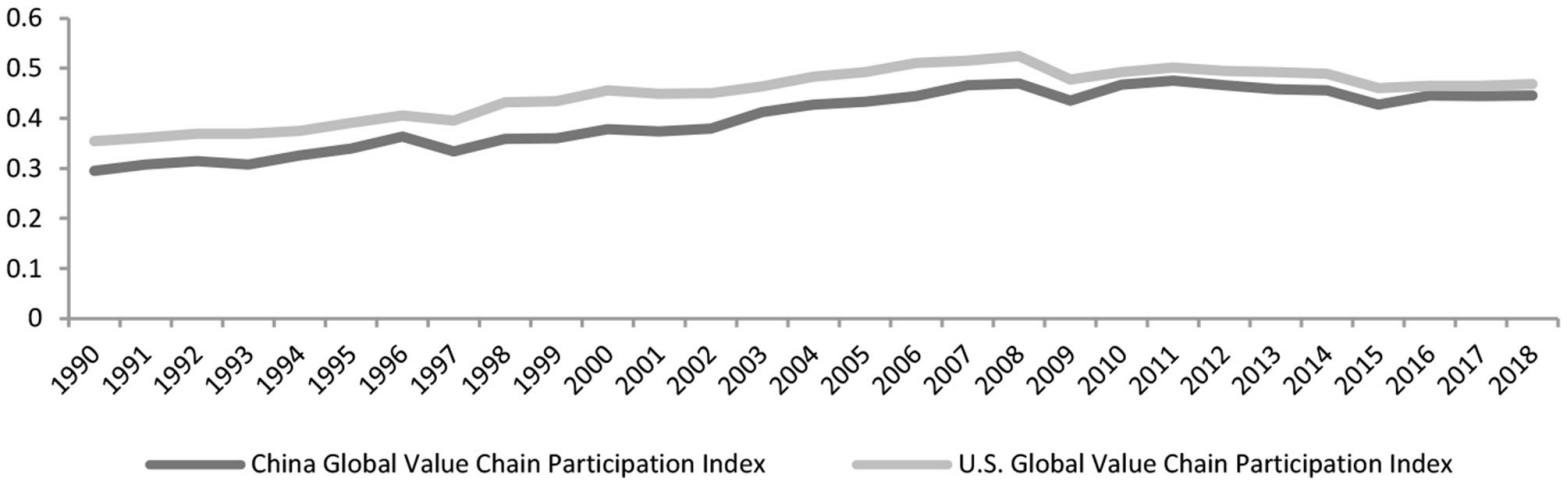
China and U.S. Global value chain participation index. Data source: UNCTAD-Eora GVC.

The post-pandemic GVCs will have three characteristics in the coming post-pandemic era as the impact of the COVID-19 pandemic on the structure of the Chinese value chains tends to be long and far-reaching. First, they're shortening at an increasingly decelerated pace. Second, geopolitical interests will cause GVC reconfiguration and a shift to security considerations from economic considerations. Third, the GVCs will take a turn to localization and regionalization. The following paragraphs provide more specifics. The combination of the pandemic with the U.S.-China trade dispute shortens the GVCs. According to UNCTAD-Eora GVC Database, the participation of China and the U.S. the GVC had topped before the 2008 financial crisis. Their 2008 GVC participation stood at 61%, 13 percentage points from 1990 (48%). Following the financial crisis, their GVC participation dropped to 57% in 2018 (Figure 7).

The radical differences between the U.S. and China in institutions have been driving the trade friction into tariff war (34). A substantive change of the U.S.-China relations has shaken mutual trust. Other major developed economies, under the influence of the U.S. policy change, take actions to reduce their reliance on China's manufacturing sector (35). In the post-pandemic era, it is likely that the major economies make attempt to withdraw their investments in the manufacturing sector of China. As investment withdraw is expected to take place in the post-pandemic era, it's expected that the GVCs will become shorter, more scattered and more localized, which makes China exposed to long-term challenges of GVC relocation.

## Models and the Index System

This part focuses on how to link the GTAP model with the location in division of labor in the GVCs measured based on the TiVA statistical method. Forward GVC participation, backward GVC participation and location in division of labor in the GVCs are constructed to effectively investigate the pandemic's impact on GVC reconfiguration in regard to Chinese manufacturing.

The GTAP model is a global multi-regional computable general equilibrium (CGE) model developed by Purdue University. In regard to application, the database is presented as Eora global multi-region input-output (MRIO) tables with global coverage. The most updated version is GTAP 10 which provides the data about 141 countries and 65 industries, accounting for 98% of the global GDP and 92% of the world's population and covering the world's major economies and segments. Compared with dynamic stochastic general equilibrium (DSGE), the CGE model transmits the external impact through the global multi-region MRIO tables, hence the likeness to the real world. The CGE model finds very wide application in FTAs and government policy simulation. It can be used for the general equilibrium study of such fields as trade, energy, agricultural and tax.

In regard to the division of labor in the international production network, the most common measure of the value chain is the TiVA statistical method which, by combining the traditional Customs statistics with the value added statistics, works out the value added for a single good generated at each stage of the production chain, from raw materials to a final good. Therefore, this paper refers to the decomposition method proposed by Wang et al. (31) and classifies a country's production activities into cross-border non-GVCs and non-cross-border GVCs. At the same time, the paper precisely assesses participation of Chinese manufacturing and its level in division of labor from three perspectives, i.e., forward GVC participation, backward GVC participation and China's level in GVC division of labor.

Wang et al. (31) classified the production activities of a country into purely domestic production activity, traditional international trade, simple GVC activity and complex GVC activity.

(1) $$\begin{array}{r} \hat{V}BŶ=\hat{V}L{Ŷ}^{D}+\hat{V}L{Ŷ}^{F}+\hat{V}LA^{F}\hat{X}=\hat{V}L{Ŷ}^{D}+\hat{V}L{Ŷ}^{F}+\hat{V}LA^{F}BŶ\\ =\hat{V}L{Ŷ}^{D}+\hat{V}L{Ŷ}^{F}+\hat{V}LA^{F}L{Ŷ}^{D}+\hat{V}LA^{F}\left(BŶ-L{Ŷ}^{D}\right) \end{array}$$

Where the sum of the columns indicates the direction of the segment value added of the various countries.

(2) $$va^{\prime }=\hat{V}BY=\underset{\left(1\right)-V\_D}{\underset{︸}{\hat{V}LY^{D}}}+\underset{\left(2\right)-V\_RT}{\underset{︸}{\hat{V}LY^{F}}}+\underset{\left(3a\right)-V\_GVC\_S}{\underset{︸}{\hat{V}LA^{F}LY^{D}}}+\hat{V}LA^{F}\underset{\left(3b\right)-V\_GVC\_C}{\underset{︸}{\left(BY-LY^{D}\right)}}$$

Where the aggregate of the ranks indicates the source of the segment value added of the various countries.

(3) $$Y^{\prime }=VB\hat{Y}=\underset{\left(1\right)-Y\_D}{\underset{︸}{VL{\hat{Y}}^{D}}}+\underset{\left(2\right)-Y\_RT}{\underset{︸}{VL{\hat{Y}}^{F}}}+\underset{\left(3a\right)-Y\_GVC\_S}{\underset{︸}{VLA^{F}L{\hat{Y}}^{D}}}+\underset{\left(3b\right)-Y\_GVC\_C}{\underset{︸}{VLA^{F}(B\hat{Y}-L{\hat{Y}}^{D})}}$$

In equation (3), $\underset{\left(1\right)-Y\_D}{\underset{︸}{VL{\hat{Y}}^{D}}}$ means domestic content of the locally consumed final goods (LCFG), not including foreign value added (FVA); $\underset{\left(2\right)-V\_RT}{\underset{︸}{\hat{V}LY^{F}}}$ means domestic content of exports (DCE) which can be seen as traditional trade; $\underset{\left(3a\right)-Y\_GVC\_S}{\underset{︸}{VLA^{F}L{\hat{Y}}^{D}}}$ means trade partner-sourced content of LCFG (PLCFG); and $\underset{\left(3b\right)-Y\_GVC\_C}{\underset{︸}{VLA^{F}\left(B\hat{Y}-L{\hat{Y}}^{D}\right)}}$ means imports content of exports (ICE). Equation (2) and Equation (3) can be divided into four addends. The first addend on the right of the equation means DVA used to satisfy final domestic demand (FDD; not including FVA), while the second addend means DVA used to satisfy final foreign demand (FFD) and can be seen as traditional trade. Equation (1) and Equation (2) differ in that in the former, $\underset{\left(1\right)-V\_D}{\underset{︸}{\hat{V}LY^{D}}}$ and $\underset{\left(2\right)-V\_RT}{\underset{︸}{\hat{V}LY^{F}}}$ mean the aggregates of value added of the downstream value chain activities of a country's specified sector, and in the latter, $\underset{\left(1\right)-V\_D}{\underset{︸}{\hat{V}LY^{D}}}$ and $\underset{\left(2\right)-V\_RT}{\underset{︸}{\hat{V}LY^{F}}}$ mean the sector's value added which contains value added of all upstream sectors.

**Figure 8:**
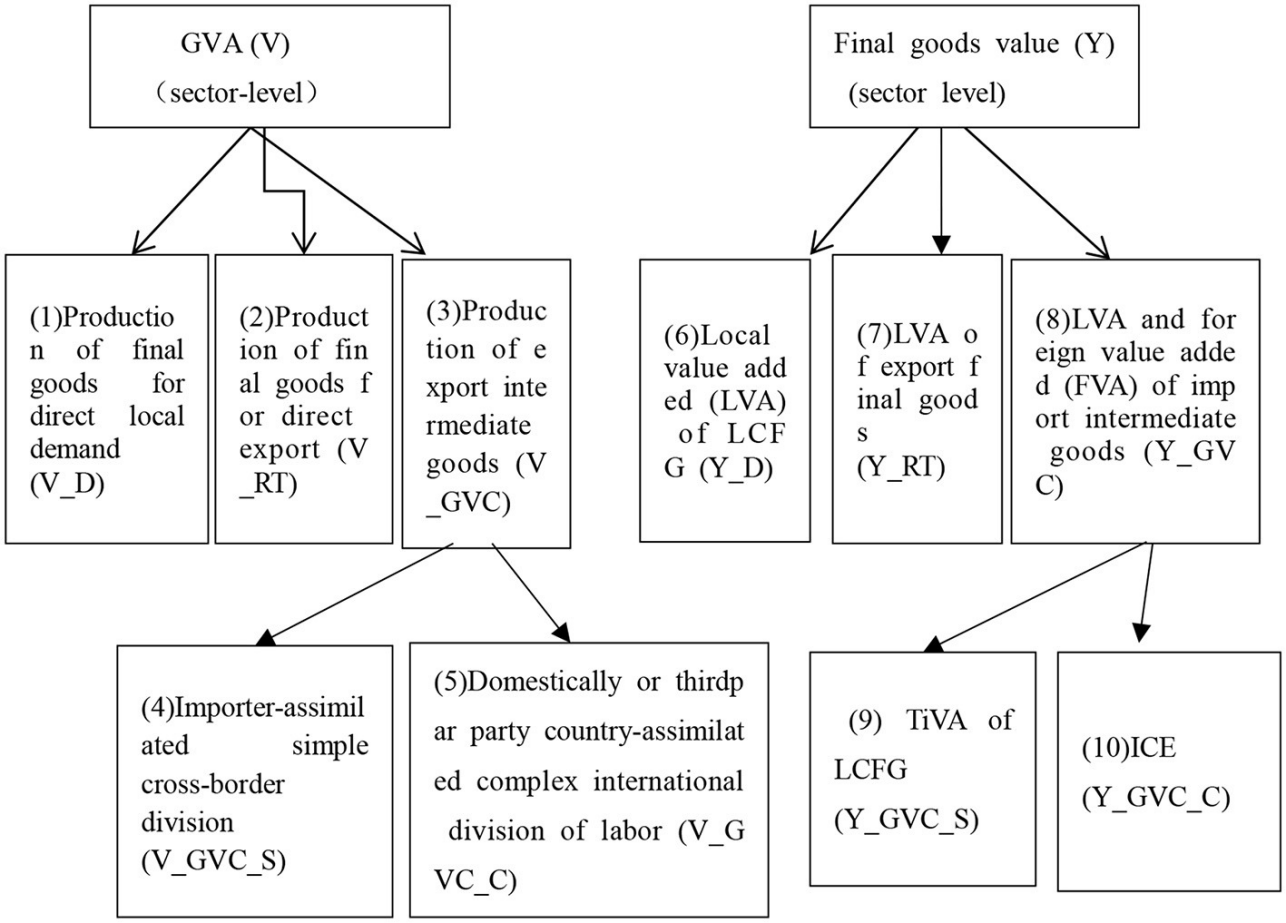
WWYZ decomposition. The left and right drawings show the decomposition of gross value added (GVA) from the perspective of forward industrial linkage and backward industrial linkage, respectively [Source: Wang et al. (31)].

Figure 8 shows the decomposition model in detail. Four types of state-sector production activities can be identified from the perspective of whether forward industrial linkage or backward industrial linkage.

The GVC Participation index measures the level of a specified sector of a country in the value chains by calculating the ratio of the sum of indirect value added (IVA) exports and FVA exports, divided by gross exports. Therefore, forward GVC participation and backward GVC participation can be expressed in terms of Equation (4) and Equation (5), respectively.

(4) $$\begin{array}{r} GVCPt_{f}=\frac{PLv\_GVC\_S}{va^{\prime }}+\frac{PLv\_GVC\_C}{va^{\prime }}=\frac{\hat{V}LA^{F}BY}{va^{\prime }} \end{array}$$

(5) $$\begin{array}{r} GVCPt_{b}=\frac{PLy\_GVC\_S}{Y^{\prime }}+\frac{PLy\_GVC\_C}{Y^{\prime }}=\frac{VLA^{F}BŶ}{Y^{\prime }} \end{array}$$

Forward GVC participation means the GVC-included share of an industry (or sector) of a country or region and reflects the capability of providing intermediate goods for the GVCs. Backward GVC participation means the contribution of domestic and foreign factors of production which participate in global production activities to the final goods value added of the country.

Meanwhile, based on the GVC division of labor method constructed by Koopman et al. (36), the paper measures the level of the division of labor in the GVCs by introducing Equation (4) and Equation (5). See the following equation for details.

(6) $$\begin{array}{r} GVCPS=\ln\left(1+GVCPt_{f}\right)-ln\left(1+GVCPt_{b}\right) \end{array}$$

Equation (6) presents the level of a specified sector of a country in the GVC division of labor. The higher the level, the closer to the upper-end of the GVCs. Besides, by referring to the methodology of Wang et al. (31), the paper trims the impact of traditional trade and purely domestic production in order to reflect the level of division of labor in the GVCs more precisely.

The standard GTAP model fails to be linked directly with the TiVA decomposition model developed by Wang et al. (31) for several database matching considerations. The first is data form. The GTAP database must be constructed based on the world input-output database (WIOD) tables. An obvious difference of the database from an I-O table is that the former must be leveled and processed in order to be constructed. Considering the difference between the GTAP database and the production decomposition database established by Wang et al. (31), the paper introduces the method developed to convert the GTAP data into the global MRIO tables. The second matching problem is imports distribution. The GTAP model can only simulate the gross trade value of different trade goods at the national level and can't depict in detail the distribution of the imports among different intermediate users and end users in the importing countries. The database, when decomposed with the KWW method (2017), must depict the distribution proportion of different trade goods of different importing countries. Therefore, the paper introduces fixed proportions, i.e., using distribution coefficient, to the global MRIO model constructed by Johnson and Noguera (37), Meng et al. (38), and Ni and Xia (39), to improve the linkage defect; that is to say, the assumption is that the proportion of an imported good consumed by the different users of a country is equal to the distribution proportion of the production and consumption structure of its domestic counterpart (40).

Considering the inadequacy of current technological and data support, the assumption above is made and the following steps are taken. First of all, use the GTAP model is used for policy simulation for the COVID-19 pandemic. Second, convert the pre-policy and post-policy GTAP simulation results to I-O data in WIOD. Third, based on the TiVA decomposition method proposed by Wang et al. (31), the pre-simulation and post-simulation data in WIOD are decomposed to work out the pre-pandemic and post-pandemic TiVA. Then the policy effect is measured in regard to the impact on China's GVC participation and level of division of labor.

## Database and Scenarios

The paper conducts a quantitative analysis based on the GTAP 10 database. The global economy is divided into 141 countries and districts, each having 65 sectors. In order to better simulate the global transmission of the pandemic and measure the impact on GVC reconfiguration at different levels of prevalence, the paper divides the 141 economies into three groups, namely China, developed countries (including the United States, Europe, Japan and South Korea) and other countries, and consolidates the 65 sectors as 46 sectors.

Because the GTAP 10 database takes 2014 as the base period, this paper uses the approaches developed by Walmsley et al. (41) to extend the database to 2020. As a basic solution, the paper introduces the method of Zhou and Zhang (40) to adjust macroeconomic variables (e.g., unskilled & skilled labor, capital, population and GDP) based on CEPII-sourced global forecast data. Notably, the paper adjusts the 2015–2020 data as appropriate in order to ensure data authenticity and database load balancing.

The COVID-19 pandemic broke out first in China, then proliferated to the developed countries and spread increasingly to other countries, including ASEAN countries. In order to systematically simulate the pandemic's impact on GVC participation of the countries and their level of division of labor, the paper the classification approach based on transmission characteristics. Based on the epidemiological theory, Cao et al. (42) characterized the cumulative curve of infection with the logistic curve. The epidemiological study of McKibbin and Fernando (9) assumes that when the pandemic is on a moderate scale, a small scale and a large scale, government spending increase by 0.5, 1.3, and 2.6%, respectively, and that labor supply decreases by 3.4, 7, and 14%. On this basis the paper makes an in-depth computation of national economy fluctuation (the data is provided by National Bureau of Statistics).

Considering the above theory and assumption and the severity of the transmission in the world, four scenarios are established. For the purpose of more credibility and convenience, the paper assumes that the stages of transmission have a deterministic effect on the pandemic's impact on government spending, resident spending and labor supply. Then we use S1, S2, S3, and S4 to represent the simulation results of the above four scenarios

Scenario 1 (S1): The pandemic, in its initial stage, has been relatively prevalent in China, but the government takes effective control measures and prevents it from transmitting to the foreign countries on a large scale.

Scenario 2 (S2): The pandemic begins to transmit to such developed countries as Japan, South Korea, Europe and the U.S. where the pandemic is on a small scale and is more serious than in other countries where the pandemic is on a moderate scale. In China, forceful control measures taken by the government enable the survival of large-scale stage, transforming the pandemic into a small-scale one.

Scenario 3 (S3): In China, the pandemic has been largely brought under control and transforms from a small scale to a moderate scale, while the pandemic has evolved into a full-scale one in such developed economies as Europe and the U.S., entering a large-scale stage; at the same time, the pandemic has transformed from a small scale to a moderate scale.

Scenario 4 (S4): In China the pandemic has been brought under full control. In the developed economies the pandemic has transformed from a large-scale to a small-scale one, while the other countries have entered a large-scale stage.

Besides, in order to ensure model stability and validity, the model should undergo homogeneity & validity testing and calibration. The calibrated model has very good stability and validity, hence its high reliability. Therefore, the paper, based on the calibrated model, further simulates the pandemic's effect on value chain reconfiguration and is linked to the TiVA decomposition model proposed by Wang et al. (31) for an analysis of pandemic's impact on China's GVC participation in GVC reconfiguration.

## Interpretation of Simulation Results

The paper simulates and dissects how the likelihood of the spreading COVID-19 pandemic would impact on the world economy. Four scenarios relevant to the four stages of transmission are presented in which the impact on labor supply, consumer spending and fiscal spending is analyzed for the purpose of defining how the pandemic impacts on import & export, trade situation, GDP growth rate and welfare policy. Table 2 provides more details.

**Table 2:** The pandemic's impact on China and other economies.

**Scenario**	**Country**	**Export changes (%)**	**Import changes (%)**	**Net export (million**	**Trade situation**	**GDP growth**	**Social welfare**
				**dollars)**	**(%)**	**rate (%)**	**changes (million**
							**dollars)**
Scenario 1	China	−6.78%	−6.20%	−26798.52	1.34	−4.82	−767011.29
	Developed countries	−0.21	−0.45	19267.29	0.26	0.03	34591.89
	Rest of the world	0.03	−0.52	−8267.73	−0.46	0.02	−29204.68
Scenario 2	China	−4.13	−1.85	−45080.88	1.06	−1.69	−295927.23
	Developed countries	−2.38	−4.32	252729.65	0.25	−4.27	−1851189.20
	Rest of the world	−2.81	−0.97	−97646.85	−0.65	−1.59	−390548.16
Scenario 3	China	−3.88	1.93	−78833.72	1.78	1.63	160086.25
	Developed countries	−3.72	−7.91	619051.44	0.19	−7.58	−3574558.58
	Rest of the world	−5.58	−1.96	−338217.75	−0.93	−3.26	−884977.19
Scenario 4	China	−4.49	2.96	−24992.44	1.35	3.12	348091.56
	Developed countries	−3.369	−4.31	158574.27	0.36	−4.27	−1628015.51
	Rest of the world	−4.51	−6.26	56418.15	−1.01	−6.76	−1857519.26

First, the pandemic situation is analyzed in regard to the impact the countries sustain in import & export and trade. There appears to be a major difference among the economies in gross foreign value and trade situation. While we see trade improved in China and the developed countries to some degree, there's a downward trend elsewhere in the world. China has achieved improvements in trade primarily because of the rise in domestic labor cost drives up exports price and therefore trade. The developed countries have also undergone trade improvements to some degree because of price elasticity of exported goods. However, the other countries see trade worsening under the impact of factor price changes.

Second, the pandemic is assessed in regard to its impact on the GDP of the economies. In the context of Scenario 1, the simulation confirms a 4.82% GDP drop. Anyhow, the outbreak of the pandemic in China has no significant impact on the developed countries and other economies, hence a minor spillover effect. In Scenario 3, the outbreak of the disease in the developed economies drives the GDP down by 7.58%, compared with Scenario 4 where the disease drives down the GDP elsewhere in the world by 6.76%.

Table 2 shows in the last column the pandemic's influence on social welfare. Overall, the negative impact of the spreading disease on social welfare .proves to be relatively significant, with China sustaining a loss of around $767 billion in social welfare in Scenario 1, the developed countries sustaining a loss of around $3.57 trillion in Scenario 3, and the other economies sustaining a loss of around $1.86 trillion in Scenario 4.

All the economies, including China, have suffered an economic impact to a varying degree, which is particularly heavy in regard of trade, GDP and social welfare of the developed economies and other economies than China.

The paper measures GVC participation of Chinese manufacturing and the level of division of labor from three perspectives, i.e., forward GVC participation, backward GVC participation and GVC division of labor, based partly on the method of Wang et al. (31). In Table 3, the outcome indicates a major difference between China and its various trade partners in regard to GVC participation and level of the division of labor.

**Table 3:** The impact of the pandemic on division of labor in GVC participation of China and its trade partners.

**Scenario**	**Country**	**Forward GVC participation**	**Backward GVC participation**	**GVC division of labor**
Scenario 1	The U.S	−1.19%	−1.08%	−0.63%
	Other developed countries	−0.48%	−0.42%	−0.35%
	Rest of the world	−0.02%	−0.12%	0.01%
Scenario 2	The U.S	0.13%	0.81%	−0.36%
	Other developed countries	0.08%	0.32%	−0.09%
	Rest of the world	−0.81%	−0.99%	−0.24%
Scenario 3	The U.S	2.61%	−1.62%	3.79%
	Other developed countries	2.74%	−1.51%	3.43%
	Rest of the world	0.31%	0.16%	0.15%
Scenario 4	The U.S	3.46%	1.27%	2.68%
	Other developed countries	1.79%	0.57%	1.23%
	Rest of the world	2.95%	−2.25%	5.23%

In the context of Scenario 1 where the pandemic is prevalent in China, the U.S.–Chinese trade friction combined with the disease results in a great fall in exports to the U.S. and a fall in imports. As China loses the shares of forward participation, backward participation in the U.S. economy and falls in the GVC level of division of labor, part of the low-end manufacturing industry is quickly redirected to the Southeast Asia; at the same time, the U.S. withdraws part of its value chain back out of China.

In Scenario 2, the pandemic has worsened in the developed countries into a small-scale one. While the other countries face a moderate-scale stage, forceful containment steps implemented in China alleviate the stress and bring the disease into a small-scale stage. Compared with Scenario 1, the gradually healing Chinese economy climbs up on the GVC level of division of labor, reversing the plummeting trend in both forward GVC participation and backward GVC participation.

In Scenario 3, when the pandemic enters the large-scale prevalent stage in such developed countries as Europe and the U.S. and leads to a stall in economic activities, they turn to China for medical supplies as the Chinese economy already begin gradual recovery. As China resumes trading with the developed economies, e.g., Europe and the U.S., the country's GVC participation level improves in division of labor.

In Scenario 4, China brings the disease under full control, the developed countries enter the small-scale stage and the other countries slide into massive prevalence. In regard to either the developed countries or the other economies, China's forward and backward participation in the GVC division of labor improves to some extent. In the meantime, the prevalence of the pandemic in other countries causes a slump in goods and service purchases by China, therefore resulting in falling backward GVC participation in other economies.

### The Impact of Pandemic on Various Sectors in Regard to Value Chain Reconfiguration

This paper is concentrated mainly on how the pandemic affects six sectors of Chinese economy in regard to the GVCs in Scenario 3. The six export sectors include agriculture, textile, automotive & parts, electromechanical equipment, computer goods and transport (Table 4). Seen from the perspective of sectoral heterogeneity, the pandemic impacts vary remarkably on different industries in regard to forward and backward GVC participation as well as GVC division of labor. In Scenario 3, the U.S.-led developed economies suffer relatively heavy impacts in terms of GVC participation and level of division of labor. China suffers relatively heavy impacts in agriculture, textile and transport in regard to forward GVC participation, a symbol that compromises the capacity of supply of primary or intermediate goods to other countries. Still, economic resumption drives China to take the lead and contribute to robust growth, which furthers its backward GVC participation.

**Table 4:** The pandemic's impact on different Chinese manufacturing sectors in regard to GVC reconfiguration.

** 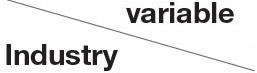 **	**Country**	**Agriculture**	**Textile**	**Automotive & parts**	**Electromechanical equipment**	**Computer goods**	**Transport**
Forward GVC	The U.S	−2.76%	−5.21%	−2.63%	4.36%	4.21%	−5.81%
participation	Other developed countries	−2.21%	−4.42%	−1.99%	3.51%	3.87%	−4.29%
	Rest of the world	−0.93%	0.56%	−0.83%	2.47%	3.02%	−0.29%
Backward GVC	The U.S	−0.13%	−0.82%	−1.65%	−0.61%	−0.68%	−2.92%
participation	Other developed countries	−2.18%	−3.02%	−1.25%	−0.23%	−0.27%	−2.85%
	Rest of the world	−0.81%	−0.04%	−0.52%	0.61%	0.56%	−0.10%
GVC division	The U.S	−2.63%	−4.32%	−0.78%	4.93%	4.88%	−2.84%
of labor	Other developed countries	−1.98%	−1.31%	−0.73%	3.89%	3.96%	−1.93%
	Rest of the world	−0.53%	0.94%	−0.27%	1.91%	2.24%	0.58%

Overall, the pandemic is proven to reconfigure GVC participation and division of labor. Pretty good disease control policy causes China to be the first of all countries to recover economy, thereby furthering its level of division of labor in GVC participation in regard to in electromechanical equipment, computer goods and other sectors where it commands global competitive advantages.

## Conclusion and Policy Advice

The outbreak of the pandemic brings impacts to China's level of division of labor on the GVCs, therefore contributing to China's forward GVC participation and furthering the level of division of Chinese manufacturing on the GVCs, although the impact varies greatly in different economic sectors.

The conclusion proposed in the paper is of great policy concern. First, the Covid-19 should be considered not only as a challenge but also an opportunity to actively promote multilateral interaction and build a regional value chain led by China, Japan and South Korea. China should play a leading role in the negotiations on the Regional Comprehensive Economic Partnership (RCEP), promote the building of a high-quality free trade area, strengthen cooperation with neighboring countries, and put in place a regular dialogue mechanism on supply chain security. At the same time, China should combine global value chain cooperation with the construction of “Belt & Road” Initiative, encouraging involved countries to strengthen the construction of supply chain system. Second, it is pointed out that in-depth structural reform should be carried out and measures taken in an effort to retain the foreign investors. At the present time, the Chinese government has implemented numerous policies to retain the foreign investors and relieve the stress of the pandemic on them. However, the foreign enterprises seek for more fundamental changes in the Chinese market, including more transparency, predictability and equality concerning in regard to regulation procedures. Therefore, more measures should be taken to boost innovation and create a competitive, business-friendly environment. Third, China should take more proactive actions in response to supply chain reconfiguration while implementing the strategy of overseas investment in manufacturing. Based on a short-term perspective, it is vitally important for China to take advantage of the opportunities from global economy recovery. Policies should be made to retain foreign investors in China and stabilize the bilateral trade relations. Based on a long-term perspective, China should make innovations of its own. As the Chinese enterprises improve innovation capability, there will be a downtrend in core technological dependence on the U.S. and an uptrend in delivering goods and services in place of import. At the same time, China should step up efforts to promote M&As and corporate reorganizations as part of the Belt and Road Initiative, add more to the GVCs, and increasingly consolidate the pivotal role of a world-class economy.

## Data Availability Statement

Publicly available datasets were analyzed in this study. This data can be found at: https://comtrade.un.org.

## Author Contributions

YS: conceptualization, methodology, formal analysis, writing—original draft preparation, and funding acquisition. XH: data processing, formal analysis, and writing—original draft preparation. YH: data processing and formal analysis. ZL: conceptualization, project management, writing–review and editing, and funding acquisition. All authors contributed to the article and approved the submitted version.

## Conflict of Interest

YH was employed by the company NPD Information Consulting (Shanghai) Co. The remaining authors declare that the research was conducted in the absence of any commercial or financial relationships that could be construed as a potential conflict of interest.
